# The synergistic antimicrobial effects of novel bombinin and bombinin H peptides from the skin secretion of *Bombina orientalis*

**DOI:** 10.1042/BSR20170967

**Published:** 2017-09-28

**Authors:** Jie Xiang, Mei Zhou, Yuxin Wu, Tianbao Chen, Chris Shaw, Lei Wang

**Affiliations:** Natural Drug Discovery Group, School of Pharmacy, Queen’s University Belfast, 97 Lisburn Road, Belfast BT9 7BL, Northern Ireland, U.K.

**Keywords:** antimicrobial, peptide, skin secretion, synergism, selectivity

## Abstract

Bombinin and bombinin H are two antimicrobial peptide (AMP) families initially discovered from the skin secretion of *Bombina* that share the same biosynthetic precursor-encoding cDNAs, but have different structures and physicochemical properties. Insight into their possible existing relationship lead us to perform the combination investigations into their anti-infectious activities. In this work, we report the molecular cloning and functional characterization of two novel AMPs belonging to bombinin and bombinin H families from secretions of *Bombina orientalis*. Their mature peptides (BHL-bombinin and bombinin HL), coded by single ORF, were chemically synthesized along with an analogue peptide that replaced L-leucine with D-leucine from the second position of the N-terminus (bombinin HD). CD analysis revealed that all of them displayed well-defined α-helical structures in membrane mimicking environments. Furthermore, BHL-bombinin displayed broad-spectrum bactericidal activities on a wide range of microorganisms, while bombinin H only exhibited a mildly bacteriostatic effect on the Gram-positive bacteria *Staphylococcus aureus*. The combination potency of BHL-bombinin with either bombinin HL or bombinin HD showed the synergistic inhibition activities against *S. aureus* (fractional inhibitory concentration index (FICI): 0.375). A synergistic effect has also been observed between bombinin H and ampicillin, which was further systematically evaluated and confirmed by *in vitro* time-killing investigations. Haemolytic and cytotoxic examinations exhibited a highly synergistic selectivity and low cytotoxicity on mammalian cells of these three peptides. Taken together, the discovery of the potent synergistic effect of AMPs in a single biosynthetic precursor with superior functional selectivity provides a promising strategy to combat multidrug-resistant pathogens in clinical therapy.

## Introduction

Bombinin, one of the typical cationic antimicrobial peptides (AMPs), was first isolated from the skin secretion of the yellow-bellied toad *Bombina variegata* [1]. The nucleotide sequence analysis of bombinin-related peptides prompted the existence of a class of structurally differentiated peptides, which were named as bombinin H [2,3]. Importantly, the presence of a subtle and inconspicuous single D-amino acid (D-alloisoleucine or D-leucine) at the second position from N-terminus of bombinin H, as a consequence of post-translational modification, was observed. This type of modification may contribute to the versatile antimicrobial mechanisms of frog skin peptides, and may be beneficial in the prevention of bacterial resistance [4–6]. However, since the initial discovery of bombinin, bombinin H and D-isoform bombinin H, research have been focused on the study of the individual peptide’s antimicrobial property, instead of their synergistic potencies. Combined effects of bombinin peptides with conventional antibiotics, and their antimicrobial selectivity towards pathogens have very rarely been reported [7].

Here, we report the structural and functional characterization of two novel, linear, cationic, α-helical AMPs, initially identified in a single ORF from the skin secretion of *Bombina orientalis*. These peptides belong to the bombinin and bombinin H families. The potent synergistic relationship of the novel bombinin and bombinin H peptides highlights the significance of combinational utility of AMPs in the treatment of infections caused by drug-resistant bacteria, this continues to provide researchers with novel approaches for prospective innovation in clinical studies.

## Materials and methods

### Specimen preparation and secretion harvesting

Specimens of the oriental fire-bellied toad *B. orientalis* were obtained from a commercial supplier and raised in a specially designed vivarium until maturation, over a period of 4 months. The skin secretions were collected and lyophilized as previously described [8]. Sampling of skin secretion was performed by Mei Zhou under U.K. Animal (Scientific Procedures) Act 1986, project license PPL 2694, issued by the Department of Health, Social Services and Public Safety, Northern Ireland. Procedures had been vetted by the IACUC of Queen’s University, Belfast, and approved on 1 March 2011.

### Molecular cloning of novel bombinin and bombinin H precursor encoding cDNA from the skin secretion derived cDNA library

A 5-mg lyophilized secretion of *B. orientalis* was dissolved in 1 ml of mRNA protection buffer, the polyadenylated mRNA was obtained by using magnetic oligo-dT beads following the instructions of the manufacturer (Dynal Biotech, Wirral, U.K.), and subsequently reverse transcribed. The cDNA was subjected to 3′-RACE PCR procedure to obtain the full-length prepro-bombinin and prepro-bombinin H nucleotide sequence using a SMART-RACE kit (Clontech, Oxford, U.K.) as described by the manufacturer. For 3′-RACE reaction, a nested universal primer (NUP) (supplied with the kit) and a degenerate sense primer were designed and performed as previously reported [9,10]. The 3′-RACE reactions were performed as per previous description [11].

### Identification and structural analysis of deduced mature peptides in the skin secretions

Another 5 mg of lyophilized secretion was dissolved in 1.0 ml of 0.05/99.95 (v/v) trifluoroacetic acid (TFA)/water and clarified by centrifugation. The rp-HPLC system was fitted with an analytical column (phenomenex C-5, 0.46 × 25 cm and pheomenex C-18, 250 × 10 mm), eluting with a linear gradient formed from TFA/dd water (0.05/99.95, v/v) to TFA/dd water/acetonitrile) (0.05/19.95/80.0, v/v/v) in 240 min at 1 ml/min. The fractions were collected automatically at a minute’s intervals and effluent absorbance was continuously monitored at λ: 214 nm and λ: 280 nm. Each reverse-phase HPLC fraction was analysed with MALDI-TOF MS on a linear TOF Voyager DE mass spectrometer (Perseptive Biosystems, MA, U.S.A.) in positive detection mode using α-cyano-4-hydroxycinnamic acid as the matrix. Fractions containing peptides with molecular masses coincident with predicted mature peptides from ‘shotgun’ cloning were infused into the LCQ Fleet™ ion-trap electrospray mass spectrometer for analysis (Thermo Quest, San Jose, CA, U.S.A.).

### Peptides synthesis and purification

The two novel identified bombinin peptides and one single-residue D-isomer analogue were synthesized by Tribute® Peptide Synthesizer (Protein Technologies, Inc., Tucson, U.S.A.) with solid-phase Fmoc chemistry methodology and amide resin. Their molecular masses were analysed and confirmed by MALDI-TOF. Then, synthetic replicates were purified with rp-HPLC to obtain high purity of synthetic peptides.

### CD spectroscopy

CD spectra between 190 and 250 nm were performed on a Jasco J-815 CD spectrometer (Jasco, Essex, U.K.). The machine units of millidegrees ellipticity were converted to mean residue molar ellipticity using the following equation (*n*, the number of peptide bonds; ellipticity is the raw data from the instrument):

$$\text{θ }(\deg.{\text{cm}}^{2}.{\text{dmol}}^{-1})\;=\;\frac{\text{Ellipticity }(\text{mdeg})\;\times \;10^{6}}{\text{Path length }(\text{mm})\;\times \;\text{Peptide}\;(\text{μM})}\times \text{n}$$

The spectra were recorded at 100 nm/min in ammonium acetate (10 mM) buffer or trifluoroethanol (TFE) (50%) solution. CD measurements were performed at 20°C with 1-mm path length of cuvette. An average of three scans were collected and automated analyses for each peptide. The final predicted percentage of secondary structure was calculated using the K2D3 CD spectra web server [12].

### Antimicrobial activity and minimal biofilm eradication concentration assays

The minimal inhibitory concentrations (MICs) of the synthetic replicates of the AMPs were determined using quality control strains, the Gram-positive bacterium, *Staphylococcus aureus* (NCTC 10788), the Gram-negative bacteria *Escherichia coli* (NCTC 10418) and *Pseudomonas aeruginosa* (ATCC 27853), the yeast *Candida albicans* (NCPF 1467) and methicillin-resistant *S. aureus* (MRSA) (ATCC 12493). The reference strains of the microorganisms were initially incubated in Mueller–Hinton broth (MHB) for 16–20 h, then the bacterial cultures were diluted to obtain 1 × 10^6^ cfu/ml for the bacterial and the yeast culture to 5 × 10^5^ cfu/ml. The samples were added to obtain final concentrations from 1 to 512 mg/l. After 24-h incubation, the OD of each well was measured at 550 nm. The MIC value was measured as the minimal concentration of peptide with an OD identical with that of the negative controls [13]. Upon achieved the data from MIC assays, 10 µl of the medium from each well was taken and inoculated on to Mueller–Hinton agar (MHA) plates. After 24-h incubation, the minimum bactericidal concentrations (MBCs) and the minimum fungicidal concentration (MFC) were obtained, which were defined as the lowest concentration of peptide from which no colonies could be subsequently grown.

The minimal biofilm eradication concentration (MBECs) of the synthetic peptides were determined against *S. aureus* and performed following a standard method as shown in manufacturer’s instructions (Innovotech, U.K.). The MBEC™ P&G assay plate with specialized peg architecture designed for the formation of biofilm was used for antibiofilm susceptibility tests. The procedure of inoculation and subculturing were performed as described before. The inoculum plate was prepared by transferring 200 µl inoculum to the 96-well plate and kept in a 150-rpm moist orbital incubator for 72 h at 37°C. After which, the lid with pegs of the inoculum plate was rinsed by PBS twice, seven replicates of a serial of two-fold diluted peptides (1–512 mg/l) along with the positive/negative controls were added to corresponding wells. After incubation at 37°C for 24 h, the recovery plate was prepared by adding 200 μl recovery medium (MHB/neutralizing agents 20/0.5 (v/v)) into each well. The lid from the inoculum plate was rinsed and the placed on the recovery plate. After sonication for 30 min, the recovery plate was measured at 550 nm. The MBEC was determined as the lowest concentration with no microbial growth detected. Melittin (Sigma–Aldrich, U.K.), first isolated from honeybee (*Apis mellifera*) venom, was taken as positive control in comparison [14,15]. Both antimicrobial and biofilm eradication assays were independently performed three times.

### Kinetic time-killing assays

The kinetic time-killing assays were performed with different concentrations of peptides alone or with another checkerboard titration predicted synergistic agents. The concentration series of peptides alone or with the synergistic counterpart were added to 1.5-ml microcentrifuge tubes, which were then inoculated with a log phase culture of the test organism as described in the above section. During the incubation, 50 μl sample from each tube was removed from culture tubes at 0-, 5-, 10-, 20-, 30-, 60- and 120-min intervals for single peptides or 0-, 0.5-, 1-, 3-, 6- and 24-h intervals for synergistic pairs. After diluting serially with PBS, 50 μl of diluted samples were inoculated on MHA plates and incubated at 37°C for 24 h for colony counts. The synergistic effect was defined as equal or higher to 2 – log_10_ – cfu/ml decrease in bacterial counts compared with the effect of the most active single constituent [16]. Curves were constructed by plotting the log_10_ of cfu/ml against time.

### Haemolysis assay

Defibrinated horse erythrocytes (TCS Biosciences Ltd, Buckingham, U.K.) were prepared to produce a 4% (v/v) suspension of red blood cells in PBS by repeated washings with sterile PBS. A range of concentrations of synthetic peptides (1–512 mg/l) were incubated with red blood cell suspension samples (200 µl) at 37°C for 120 min. After incubation, the suspensions of each sample were centrifuged to obtain the final lysis of red blood cells. OD measurements of supernatants were recorded at 550 nm. Negative controls were prepared by a 2% (v/v) suspension with PBS in equal volumes (0% haemolysis), while the positive controls employed a 2% (v/v) suspension with 2% (v/v) of the non-ionic detergent, Triton X-100 (Sigma–Aldrich, U.K.) in PBS. HC_50_ was defined as the peptide concentration that caused 50% haemolysis.

### Cytotoxicity testing

The cytotoxicity of synthetic peptides on mammalian cells was examined using human microvessel endothelial cell (HMEC-1), which were cultured with MCDB 131 medium (Gibco, U.K.) supplemented with 10% FBS, 10 mM L-glutamine, 10 ng/ml EGF and 1% penicillin-streptomycin; and 5 ×10^3^ cells/well were seeded into 96-well plates. After 24-h incubation at 37°C with 5% CO_2_, 12-h serum-free starvation was performed, peptides with 10^−9^–10^−4^ M concentrations were added for 24 h treatment prior to 10 µl MTT (5 mg/ml PBS) incubation for 4 h, the growth medium was removed followed by adding 100 µl of DMSO to dissolve the formazan crystals. The absorbance was measured at 570 nm. Data from the present study were analysed by *t*test using GraphPad Prism (version 5.01). A *P*-value less than 0.05 was considered a significant difference. Negative and positive control treatments were carried out with culture medium and 1% Triton X-100 respectively. Data from the present study were analysed by one-way ANOVA with Bonferroni’s post test.

### Evaluation of combination effects of antimicrobial AMPs

A 2D checkerboard with two-fold dilutions of each AMP was used for examining the combination effects with *S. aureus*. The dissolved samples of each peptide or antibiotic agent were diluted from 4× MIC to 1/16× MIC. The series of component A were added along the row of a 96-well plate, while the columns were filled with the diluted component B. Growth control wells containing only microorganism medium and sterility control wells with only MHB medium were included. After the addition of a log-phase bacterial inoculum at 1 × 10^6^ cfu/ml, plates were incubated at 37°C for 24 h and then measured the λ at 550 nm. The combination effects were examined by calculating the fractional inhibitory concentration index (FICI) of each combination as follows:

$$\frac{\text{MIC of component A},\;\text{tested in combination}}{\text{MIC of component A},\;\text{tested alone}}\;+\;\frac{\text{MIC of component B},\;\text{tested in combination}}{\text{MIC of component B},\;\text{tested alone}}$$

After the combination ratio of the two tested compounds was confirmed, lower concentration pairs were selected to determine the FICI with more accuracy. The profile of the combination was interpreted as synergistic for FICI ≤0.5, additive for 0.5< FICI ≤4.0, and antagonistic for FICI >4.0 [17,18].

For assessing the synergetic activity of bombinin and bombinin H against the growth of mammalian cell lines, both CalcuSyn software [19] and Jin’s formula [20] were employed. Combination index (CI) plots were generated by using CalcuSyn software. A value of CI <1 represents synergy. The following formula was used in Jin’s formula : Q = Ea+b (Ea + Eb − Ea × Eb). Q is the CI; Ea+b represents the cell proliferative inhibition rate of two AMPs; Ea and Eb represents the cell proliferative inhibition rate for individual peptide. After calculation, the results Q >1.15 indicates synergy, and 0.85< Q <1.15 indicates an additive effect [19].

## Results

### Molecular cloning of skin secretion precursor cDNA encoding bombinin and bombinin H

**Figure 1:**
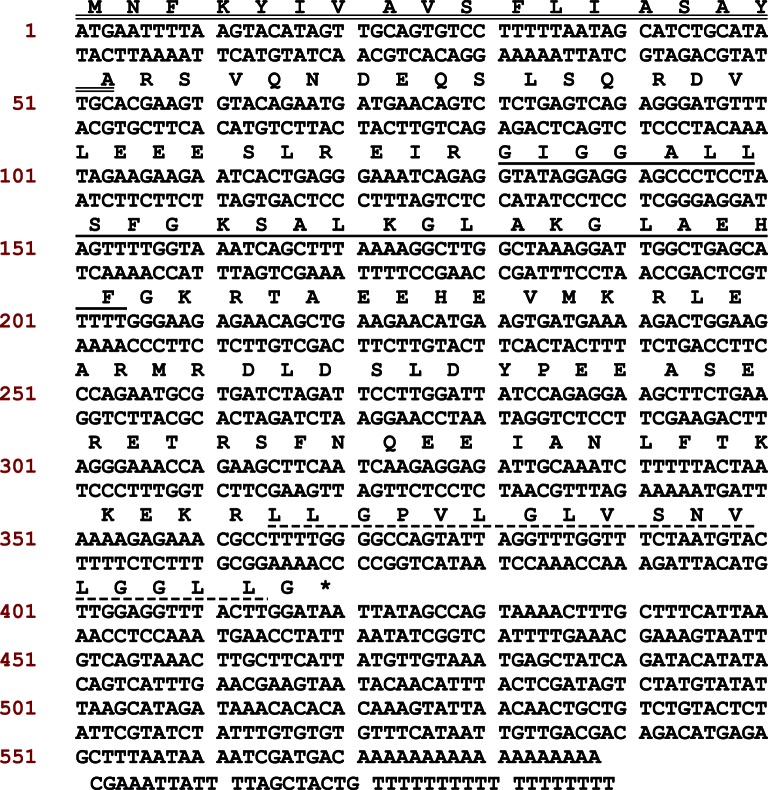
The nucleotide sequence and ORF amino acid sequence of full-length pre-probombinin and pre-probombinin H peptides encoding cDNA from the oriental fire-bellied toad, *B. orientalis* The putative signal peptide is double-underlined. The mature peptide is single-underlined for bombinin and dash-underlined for bombinin H. The stop codon is indicated by an asterisk.

The full-length biosynthetic precursor-encoding cDNAs were cloned from the skin secretion derived cDNA libraries of *B. orientalis*. The nucleotide of full ORF of the cloned precursor transcripts and its translated sequences are shown in Figure 1, which contains 139 residues and encodes a novel bombinin (BHL-bombinin) and a novel bombinin H (bombinin HL).

The nucleotide sequence of the cDNA encoding BHL-bombinin and bombinin HL precursor from the skin secretion of *Bombina orientalis*, has been deposited in the EMBL Nucleotide Sequence Database under the accession code: LT615078.

**Figure 2:**
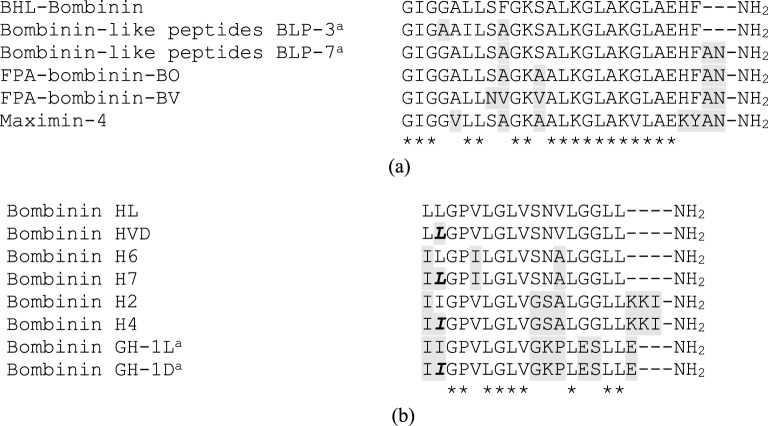
Primary structure analysis of BHL-bombinin and bombinin HL with other bombinin-related peptides. Alignment of the primary structure of the novel peptides BHL-bombinin (**a**) and bombinin HL (**b**) with other bombinin family peptides. An * (asterisk) indicates positions that have a single, fully conserved residue. Substitutions are highlighted in gray. D-amino acids are bold and italic. Gap residues are represented by dashes. ^a^ Sequences deduced from *BLP-3* and *BLP-7* genes. Abbreviation: GH, gene-derived bombinin H-like peptide.

The sequences of two novel AMPs were subjected to online BLAST program analysis with the NCBI online portal. The resulting typical primary structures were compared in Figure 2. The BHL-bombinin and bombinin HL, which were exhibited as tandem mature peptides in biosynthetic precursor in Figure 1, revealed 96 and 82% sequence identity respectively, with other bombinins identified from Bombinatoridae. The main sequence difference was indicated in the last two or three residues in the C-terminus, where BHL-bombinin is -Ala-Asn- loss and bombinin HL is truncated of -Lys-Lys-Ile- with a typical valine residue at 12th position from N-terminus (Figure 2).

### Identification and structure characterization of novel bombinin and bombinin H by rp-HPLC and MS/MS fragmentation

**Figure 3:**
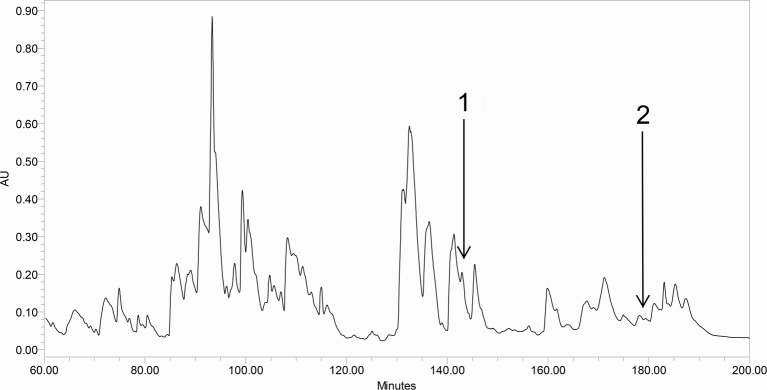
rp-HPLC spectrum of crude *B. orientalis* skin secretion. Region of reverse-phase HPLC chromatogram of *B. orientalis* skin secretion with arrows indicating the retention times of the novel BHL-bombinin (1) and bombinin HL (2). The detection wavelength was 214 nm with a flow rate of 1 ml/min in 240 min.

**Figure 4:**
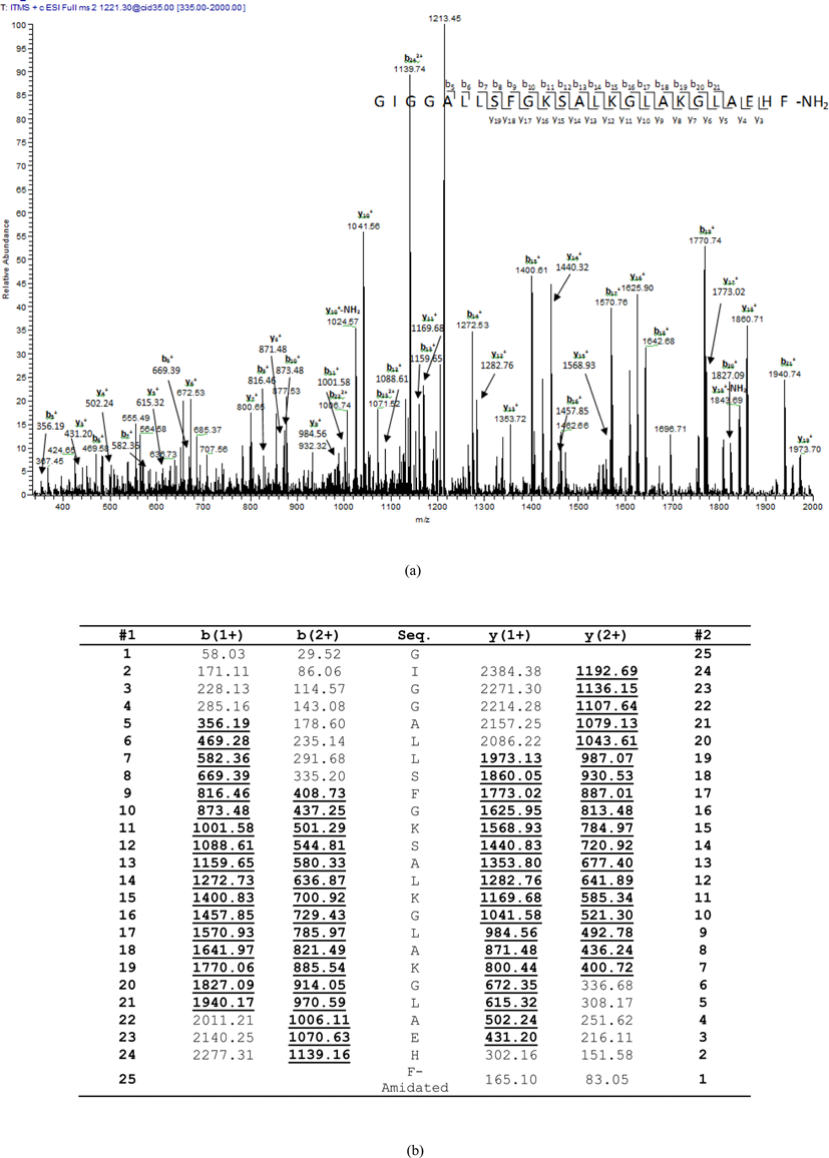
Predicted BHL-bombinin sequence from selected rp-HPLC fraction using LCQ-Fleet. Thermoquest LCQ™ fragment scan spectrum derived from ions corresponding to BHL-bombinin (**a**) and electrospray ion-trap MS/MS fragmentation dataset (**b**). Expected single- and double-charged b- and y-ions arising from MS/MS fragmentation were predicted using the MS Product program through Protein Prospector online. Truly observed ions are indicated in bold typeface and underlined.

**Figure 5:**
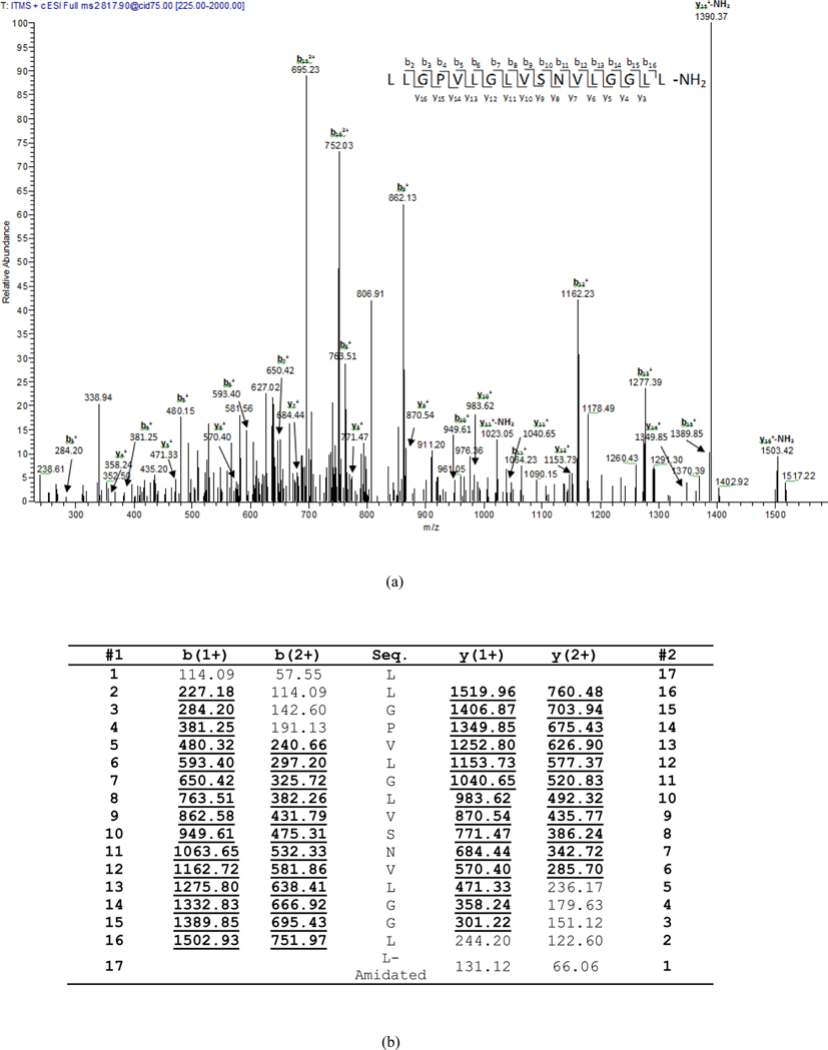
Predicted bombinin HL sequence from selected rp-HPLC fraction using LCQ-Fleet. Thermoquest LCQ™ fragment scan spectrum derived from ions corresponding to bombinin HL (**a**) and electrospray ion-trap MS/MS fragmentation dataset (**b**) Expected single- and double-charged b- and y-ions arising from MS/MS fragmentation were predicted using the MS Product program through Protein Prospector online. Truly observed ions are indicated in bold typeface and underlined.

HPLC fractions with molecular masses coincident with predictions from molecular cloning for BHL-bombinin and bombinin HL were identified (Figure 3) following detection by the ion-trap of the LCQ Fleet mass spectrometer with further testing by MS/MS fragmentation sequencing of doubly charged ions derived from frog skin secretions (Figures 4 and 5).

### CD spectra and bioinformatic analysis

The secondary structures of synthetic replicates of AMPs were investigated in 10 mM of ammonium acetate (pH 7.0, mimicking aqueous environment) and 50% TFE (mimicking the hydrophobic environment of the microbial membrane) by CD spectroscopy. As shown in Supplementary Figure S2, all the peptides displayed random coil conformations in the aqueous environment. However, the spectrums of peptides were characteristic of α-helix conformations in the presence of 50% TFE, as indicated by the presence of double-negative dichroic bands at approximately 208 and 222 nm. The web server K2D3 calculation revealed that the helical content for BHL-bombinin is 87.59%, and for bombinin HD and bombinin HL are 77.73% in 50% TFE solution.

The physiochemical parameters of novel AMPs are listed in Table 1, which not only provides evidence of the possible interactions between peptides and bacterial membrane but also gives more information on their synergistic mechanisms. The molecular masses of synthetic peptides were determined by MALDI-TOF (Supplementary Figure S1). Physiochemical parameters including charge, hydrophobic moment (μH) and hydrophobicity were determined using the Heliquest server [21]. The μH was determined by Eisenberg’s scale with a full window in Heliquest server. BHL-bombinin elicits higher cationic but much less hydrophobic properties than bombinin HL, which exhibit highly structural and physiochemical differences between the two co-encoded AMPs. These findings suggest that these two peptides may possess distinguishing roles in the interaction with microorganisms and exhibit potent antibacterial activities synergistically.

**Table 1:** Physicochemical characteristics of the novel AMPs BHL-bombinin and bombinin HL

Peptides	Theoretical Mw	Measured Mw	Hydrophobicity (H)	μH	Net charge	% helicity
**BHL-bombinin**	2441.87	2441.50	0.462	0.404	+3	87.59
**Bombinin HL**	1633.03	1633.02	0.920	0.501	+1	77.73

### Antimicrobial and haemolytic activities

The antimicrobial effects of synthetic AMPs on the growth of the tested microorganisms, and the biofilm eradication effects on *S. aureus* are illustrated in Table 2. The BHL-bombinin exhibited stronger antimicrobial activities on Gram-positive bacteria (MIC/MBC: 4 mg/l/16 mg/l) and yeast (MIC/MBC: 4 mg/l/16 mg/l) than Gram-negative bacteria (MIC/MBC: 16–64 mg/l/64–128 mg/l). In addition, BHL-bombinin was found to possess a relatively low level of haemolytic activity (0–12.6%) at the MIC determined against *S. aureus* and *C. albicans* (Supplementary Figure S3). Interestingly, BHL-bombinin displayed potent inhibitory effects (MIC: 4–16 mg/l) towards MRSA and biofilm. By contrast, the MIC values for bombinin HL and bombinin HD against *S. aureus* were 256 and 128 mg/l respectively with undetected MBCs, which were significantly less effective compared with BHL-bombinin. The selectivity indices (SIs), which represent the degree of antibacterial selectivity, are showed in Table 2, higher SI value reflecting a better selectivity towards microbial over mammalian membranes [22]. As indicated, the BHL-bombinin had a higher SI compared with bombinin HL and bombinin HD, which is in agreement with previous studies that high level of hydrophobicity may decrease the antimicrobial selectivity of α-helical peptides [23]. Additionally, compared with the melittin peptide, all the AMPs investigated in the present study exhibited 32–128-times higher SI values, which emphasizes that amphibian-derived AMPs are potential research targets for therapeutic alternatives to current antibiotics. The time-killing curves demonstrated the faster cell-killing effects of BHL-bombinin compared with the ampicillin, while the kill rates of bombinin HL and bombinin HD were relatively low (Supplementary Figure S4).

**Table 2:** The susceptibility of novel AMPs and melittin peptide against microbial strains, bacterial biofilm and their SIs against *S. aureus*

Peptides	MIC/MBC (mg/l (µM))				MIC/MFC (mg/l (µM))	MBEC (mg/l (µM))	HC_50_ (mg/l (µM))	SI^b^
	Gram-positive bacteria		Gram-negative bacteria		Fungi	*S. aureus*		
	*S. aureus*	*MRSA*	*E. coli*	*P. aeruginosa*	*C. albicans*			
**BHL-bombinin**	4 (1.6)/16 (6.6)	16 (6.6)/64 (26.2)	16 (6.6)/64 (26.2)	64 (26.2)/128 (52.4)	4 (1.6)/16 (16.6)	4(1.6)	64 (26.2)	16
**Bombinin HL**	256 (156.8)/NA^a^	NA/NA	NA/NA	NA/NA	NA/NA	NA	>512 (313.5)	4
**Bombinin HD**	128 (78.4)/NA	NA/NA	NA/NA	NA/NA	NA/NA	NA	>512 (313.5)	8
**Melittin**	8 (2.8)/16 (5.6)	32 (11.2)/64 (22.4)	16(5.6)/32(11.2)	16 (5.6)/64 (22.4)	8 (2.8)/16 (5.6)	8(2.8)	1 (0.4)	0.125

a NA, not active; no inhibition or bactericidal activity was observed using peptide concentrations up to and including 512 mg/l.

b SI is defined as the ratio of HC_50_ to MIC against *S. aureus* value (HC_50_/MIC). When no or mild haemolysis was observed at the highest concentration employed (512 mg/l), a value of 1024 mg/l was used for calculation.

**Figure 6:**
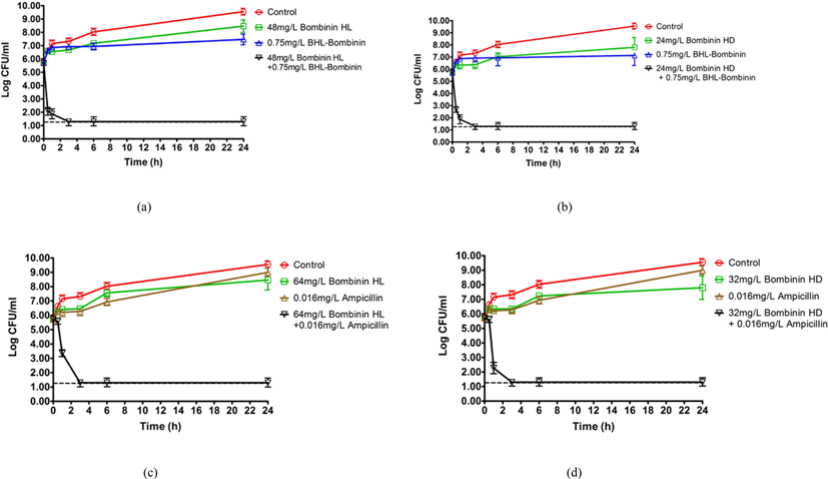
Time-killing curves for combinational treatment of peptides and antimicrobial agents against *S. aureus* Time-killing curves for combinational treatment of peptides [Bombinin HL and BHL-bombinin (**a**); Bombinin HD and BHL-bombinin (**b**)], and antimicrobial agents [Bombinin HL and ampicillin (**c**); Bombinin HD and ampicillin (**d**)] against *S. aureus*. Control (red circle/line), Bombinin HL and Bombinin HD (green square/line), ampicillin (brown triagle/line), BHL-Bombinin (purple triagle/line) and combination pairs (black inverted triagle/line) are indicated in the graphs. The detection limit is shown as a dashed line. The graphs were derived from values of three independent trials.

The combined administration of BHL-bombinin with either bombinin HL or bombinin HD revealed a synergistic antimicrobial effect against *S. aureus* (FICI: 0.375). In addition, BHL-bombinin showed additive property with classic antibiotic ampicillin (FICI: 0.75), while the novel bombinin H, either D- or L-amino isoforms, displayed synergistic activities with β-lactam and ampicillin (FICI: 0.5). The results were summarized in Table 3. The synergistic effects were further confirmed by the outcomes of time-killing assays. Figure 6a,b exhibits that the isolated *S. aureus* had a 6.16 (±0.76) log_10_ decrease in cfu/ml at 24 h when incubated with BHL-bombinin (0.75 mg/l) and bombinin HL (48 mg/l), compared with the single peptide effect. The time-killing value was 5.83 (±0.67) log_10_ for combined effects of BHL-bombinin (0.75 mg/l) and bombinin HD (24 mg/l). As shown in Figure 6c,d synergistic effects were also observed when co-administrated bombinin HL (64 mg/l) with ampicillin (0.016 mg/l) or bombinin HD (32 mg/l) with ampicillin (0.016 mg/l), which demonstrated a 7.51 (±0.97) log_10_ and 6.57 (±0.77) log_10_ decrease in cfu/ml at 24 h respectively.

**Table 3:** The combinational effects of the novel AMPs with ampicillin against *S. aureus* using checkerboard titration method

Antimicrobial treatment settings		Lowest FICI ([A]/[B] in mg/l)	Results
A	B		
BHL-bombinin	Bombinin HL	0.375 (0.75/48)	Synergistic
	Bombinin HD	0.375 (0.75/24)	Synergistic
	Ampicillin^a^	0.75 (2/0.016)	Additive
Bombinin HL	Ampicillin	0.5 (64/0.016)	Synergistic
Bombinin HD	Ampicillin	0.5 (32/0.016)	Synergistic

a The MIC values of ampicillin is 0.0625 mg/l against *S. aureus*.

### Cytotoxicity assessment of novel bombinin, bombinin H and their synergistic effect on HMECs

**Figure 7:**
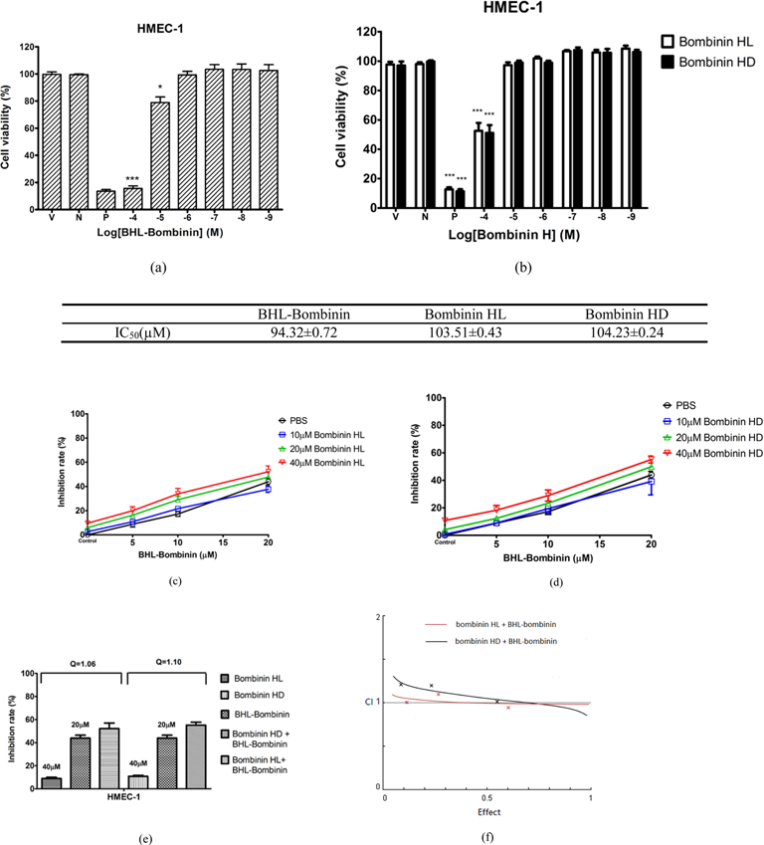
Cytotoxicity assessment of AMPs Dose-dependent antiproliferative effects of BHL-bombinin (**a**), bombinin HL and bombinin HD (**b**) against HMEC-1 after 24 h of incubation (V, N and P represent vehicle control, negative control and positive control respectively). The levels of significance are: *P<0.05; **P<0.01; ***P<0.001 compared with vehicle. The growth inhibition rate graphs of combination effects among a series concentration between BHL-bombinin with bombinin HL (**c**) or bombinin HD (**d**) against HMEC-1 after incubation for 24 h. (**e**) CI Q of the combination treatment of synergistic pairs, where Q <0.85, Q >1.15 and 0.85< Q <1.15 represent antagonism, synergy and additive effect respectively. (**f**) CI-effect plots were generated using CalcuSyn software. The points represent CI values for the combinations 5, 10, 20 µM BHL-bombinin with 10, 20, 40 µM bombinin HL or bombinin HD in a constant ratio against HMEC-1.

The antiproliferative effect data obtained from MTT cell viability assays of each peptide on HMEC-1 are represented in Figure 7a,b, and their IC_50_ values are calculated. All the AMPs tested in the present study exhibited low cytotoxicity with cell viabilities exceeding 90% up to the concentration 10^−5^ M against HMEC-1. For BHL-bombinin, at MICs (1.6–26.2 µM), 83.5–100.0% HMEC-1 remained viable. For bombinin HL and bombinin HD, they displayed relatively lower selectivity and higher cytotoxicity on HMEC-1 compared with BHL-bombinin. To identify the possible synergistic cytotoxicity between BHL-bombinin and bombinin HL or bombinin HD, the cells were cultured with combinations of these two peptides at different doses but in a constant ratio (BHL-bombinin to bombinin HL or bombinin HD: 5–10 μM, 10–20 μM and 20–40 μM respectively) for 24 h (Figure 7c,d). The combination of 20 μM BHL-bombinin with 40 μM bombinin HL inhibited cell growth of 52.21%, compared with mono-administration of BHL-bombinin (43.93%) or bombinin HL (9.63%), indicating an additive effect (CI =1.03; Q =1.06). The values for combination of BHL-bombinin and bombinin HD were CI =0.98; Q =1.10. The results revealed that the synergistic relationship was abolished with 0.85< Q <1.15 and CI ≥1 with regard to their cytotoxicity on normal mammalian cells (Figure 7e,f).

## Discussion

Different from the well-studied bioactive peptides from the amphibian Pipidae, Hylidae, Ranidae and Pseudidae families, skin secretions from *Bombina* species, remain to be investigated fully and may yield valuable promotion for drug development. The best-known constituent identified from *Bombina* skin secretions is bombesin, which leads to the subsequent identification of the mammalian homologues, gastrin-releasing peptide (GRP) and neuromedin B (NMB) as neuropeptides [24]. Among all the molecules secreted from *Bombina* species, no counterparts of the novel BHL-bombinin and bombinin HL, which are encoded by single coding region precursor, have been identified in other amphibian genera or in mammals [25].

The present study describes the molecular cloning, primary structure identification, chemical synthesis and bioactive examinations of two tandem-coded novel bombinin peptides. Since the D-isomer exists in some of the bombinin H-type molecular at the second position of their sequences, the analogue bombinin HD was designed by substituting the L-leucine at such position. CD studies revealed that the helical content of BHL-bombinin was only approximately 10% higher than bombinin HL and bombinin HD, while the hydrophobicity of BHL-bombinin is significantly lower than that of bombinin HL and bombinin HD. Therefore, all the tested AMPs in the present study were found to adapt an amphipathic α-helical conformation in a membrane mimetic environment, a feature that is essential for allowing AMPs to exert their bioactivities [26]. However, due to the diversities of their primary structures and physiochemical parameters, the functional mechanisms that they employed can be significantly different.

Synthetic BHL-bombinin were found to possess potent antimicrobial activities against *S. aureus* and *C. albicans*, but relatively lower activity against *E. coli* and *P. aeruginosa*. The MBCs for all the four tested microorganisms were approximately equal to or over four-fold of their respective MICs. Clinically, the formation of biofilm and conventional antibiotic-resistant MRSA strain are two major causes of antibiotic crisis. BHL-bombinin showed potent effects for eliminating *S. aureus* biofilm and inhibiting the growth of isolated MRSA. However, the MICs observed for wild-type bombinin HL and analogue bombinin HD, were moderately effective against *S. aureus* with undetected MBC. The antimicrobial properties of the peptides reported in the present study were further compared against melittin, which is a well-studied bee venom derived AMP [15]. BHL-bombinin exhibited similar antimicrobial activity to melittin, but weaker haemolytic activity, which indicates a better selectivity. Both bombinin H peptides possessed mild antibacterial property but higher selective antimicrobial activity compared with melittin. Following on from this, the BHL-bombinin and bombinin HL are tandem encoded in single ORF, which prompted us to speculate that their combination effect might be vital for frogs to survive in pathogen-rich environments. Of note, the combination effect between novel components and conventional antibiotics has also been proven as a promising solution to amplify the potency of antibiotics, a good example is co-amoxiclav, which enormously enhances amoxicillin potency after combined use of clavulanic acid [27]. As expected, the combination interaction of BHL-bombinin with either bombinin HL or bombinin HD showed synergistic inhibition activities against *S. aureus* (FICI: 0.375). Moreover, BHL-bombinin showed additive effect with classic antibiotic ampicillin (FICI: 0.75), while the bombinin HL and HD, displayed synergistic activities with β-lactam, ampicillin (FICI: 0.5). The results were further confirmed by time-killing assays, that the BHL-bombinin exerted higher bactericidal rate compared with ampicillin. However, the killing rates for bombinin HL and bombinin HD were diminished. The mechanism of the positive outcomes between peptides and conventional antibiotics (ampicillin in the present study) appears to be complex. The FICI as a measure of synergy employed in the present study is the best known and very basic method for evaluating the inhibitory effects of paired agents comparing the sum of their effects alone. We calculate their FICI according to the protocol for investigating their synergistic relationships at preliminary level in the present study [17,18]. For addressing the more complicated natural environment, the detailed concentration and structural relationship between peptides in this work needs a further and more systematic mechanism evaluation, which depends on the physiochemical parameters and their combination results of BHL-bombinin and bombinin HL, BHL-bombinin may have direct and selective membrane permeabilizing activity, which increases the uptake of other antibacterial agents that initiate the process to interfere with intracellular targets or enhance the effect of highly hydrophobic molecules like bombinin HL. On the other hand, either bombinin HL or bombinin HD may cause degradation of the peptidoglycan by triggering the activity of bacterial murein hydrolases, which can enhance the activity of the β-lactams [28–30].

Safety evaluations via haemolytic assay demonstrated the relatively lower SIs of both bombinin HL and bombinin HD on horse erythrocytes compared with BHL-bombinin, as a consequence, when treated HMEC-1 with the peptides using their MICs for MTT-based viability assessment, bombinin HL and HD exhibited higher cytotoxicity. The typical theory is that the increased hydrophobicity of the AMPs is associated with higher antimicrobial activity, but in contrast, the high hydrophobic peptides are associated more with stronger self-assembly, which can result in the formation of dimers or oligomers. This spatial character may in turn decrease their potential for passing through the target cell wall and bacterial membrane [31]. The high toxicity of BHL-bombinin in the present study might be mainly due to its innate character of high hydrophobicity. Additionally, the abolishment of combined effect of BHL-bombinin and bombinin H against HMEC-1 revealed their high functional selectivity. The application of combined antimicrobial agents, either with AMPs or with conventional antibiotics, is a prospective strategy to improve clinical therapy caused by multidrug resistant pathogens and decrease the side effect [32].

## Conclusion

In this project, the novel BHL-bombinin, bombinin HL and analogue bombinin HD are reported from less-studied frog species *B. orientalis*. They revealed comparable antimicrobial property individually and enhanced synergistic effect and selectivity jointly, all these inherent and robust characteristics hold significant potential to alleviate the current antibiotics crisis.

## Supporting information

**Figure S1:** MALDI-TOF (Perceptive Biosystem, Bedford, MA, USA) mass spectrum of synthetic peptide (a) bombinin HL and (b) bombinin HD and (c) BHL-bombinin. In (b), the initial neutral molecule bombinin HD [M] and metal ion adducts ([M+Na]+: 1654.35Da and [M+K]+: 1669.70Da) were observed.

**Figure S2:** CD spectra of the peptides in 10 mM ammonium acetate buffer (triangles) and 50% TFE (circles). The mean residue ellipticity was plotted against wavelength. The values from three scans were calculated as average per sample. The peptide concentrations were fixed at 100 μM.

**Figure S3:** Haemolytic activities of BHL-bombinin (diamond), bombinin HL (square), bombinin HD (triangle) and melittin (cross) following incubation with horse erythrocytes for 2 h.

**Figure S4:** Time-killing curves of peptides and ampicillin at a series of concentrations: control (red filled circle), 0.25×MIC (red unfilled circle), 0.5×MIC (green diamond), 1×MIC (blue inverted triangle), 2×MIC (purple triangle) and 4×MIC (black square) against A: *S. aureus* [(a) BHL-bombinin (b) bombinin HL (c) bombinin HD (d) ampicillin]; B: *E. coli* [(e) BHL-bombinin (f) ampicillin]; C: *C. albicans* [(g) BHL-bombinin]. The detection limit was indicated as dashed line and the graphs were derived value of three independent trials.

## Abbreviations

AMP: antimicrobial peptide
cfu: colony forming unit
CI: combination index
FICI: fractional inhibitory concentration index
μH: hydrophobic moment
HMEC-1: human microvessel endothelial cell
LCQ: liquid chromatorgraphy quadrupole
MBC: minimum bactericidal concentration
MBEC: minimal biofilm eradication concentration
MFC: minimum fungicidal concentration
MHA: Mueller–Hinton agar
MHB: Mueller–Hinton broth
MIC: minimal inhibitory concentration
MRSA: methicillin-resistant *Staphylococcus aureus*
rp-HPLC: reverse-phase high-performance liquid chromatography
SI: selectivity index
TFA: trifluoroacetic acid
TFE: trifluoroethanol
HC 50: the concentration that an antimicrobial agent kills 50% red blood cells
