# Supplementary material for: The synergistic antimicrobial effects of novel bombinin and bombinin H peptides from the skin secretion of Bombina orientalis
Source: Biosci Rep. 2017 Sep 28;37(5):BSR20170967. doi: 10.1042/BSR20170967 (PMC5634238; doi:10.1042/BSR20170967)
Supplement: Supplementary file 1 [file bsr20170967_Supp1.pdf]

Supplementary data

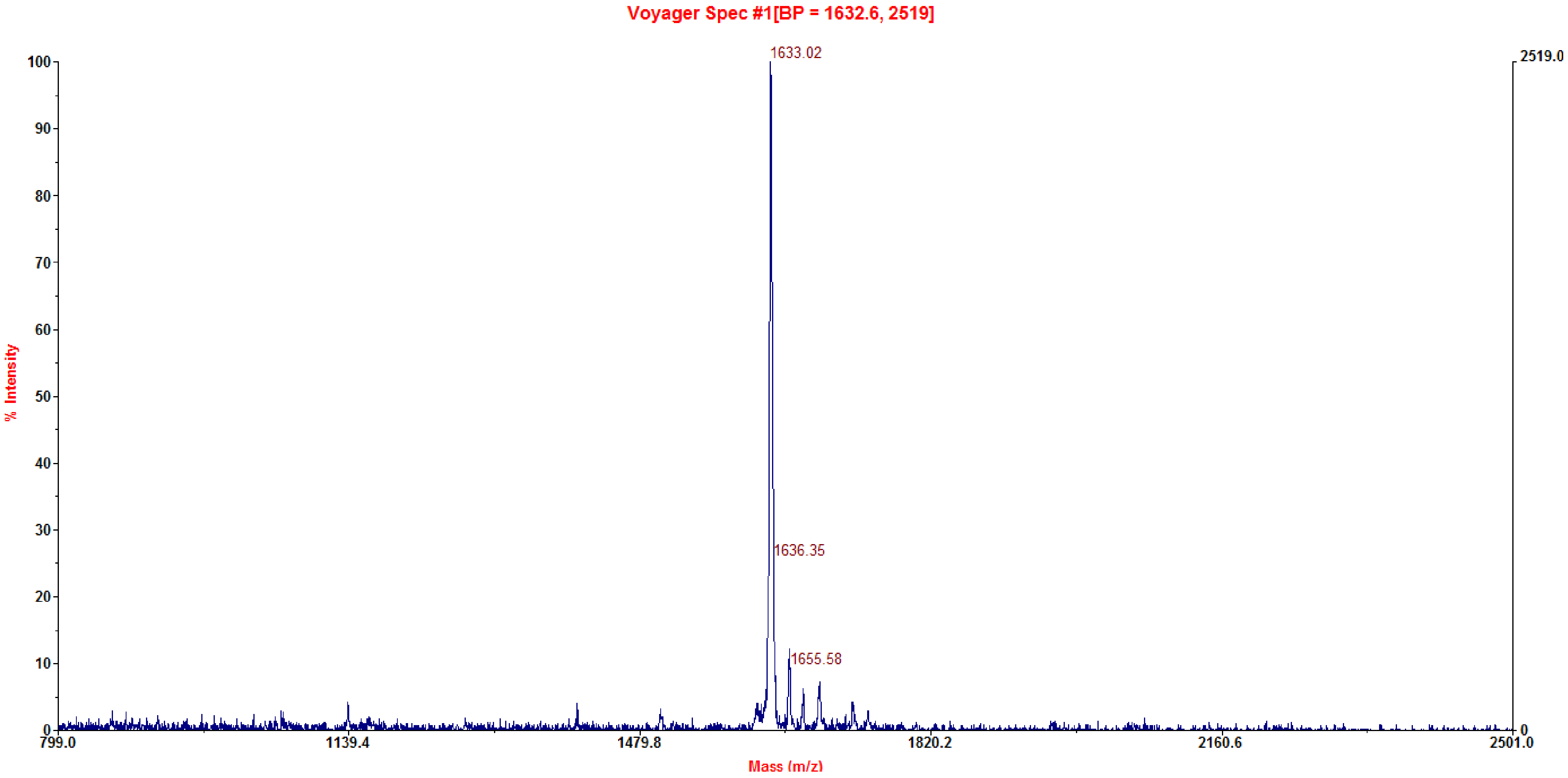

(a)

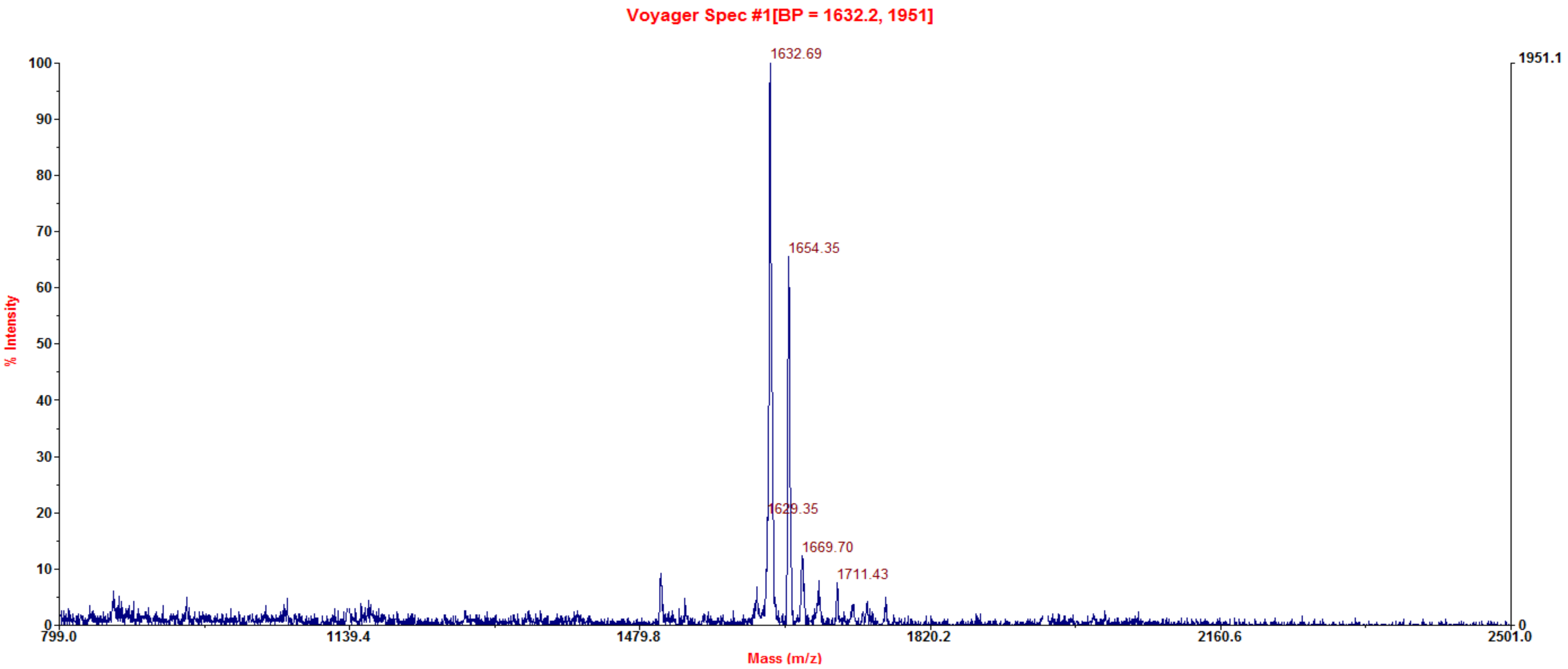
Voyager Spec #1[BP = 1632.2, 1951]
1632.69
1654.35
1629.35
1669.70
1711.43
1951.1
% Intensity
Mass (m/z)
799.0
1139.4
1479.8
1820.2
2160.6
2501.0
100
90
80
70
60
50
40
30
20
10
0
0

(b)

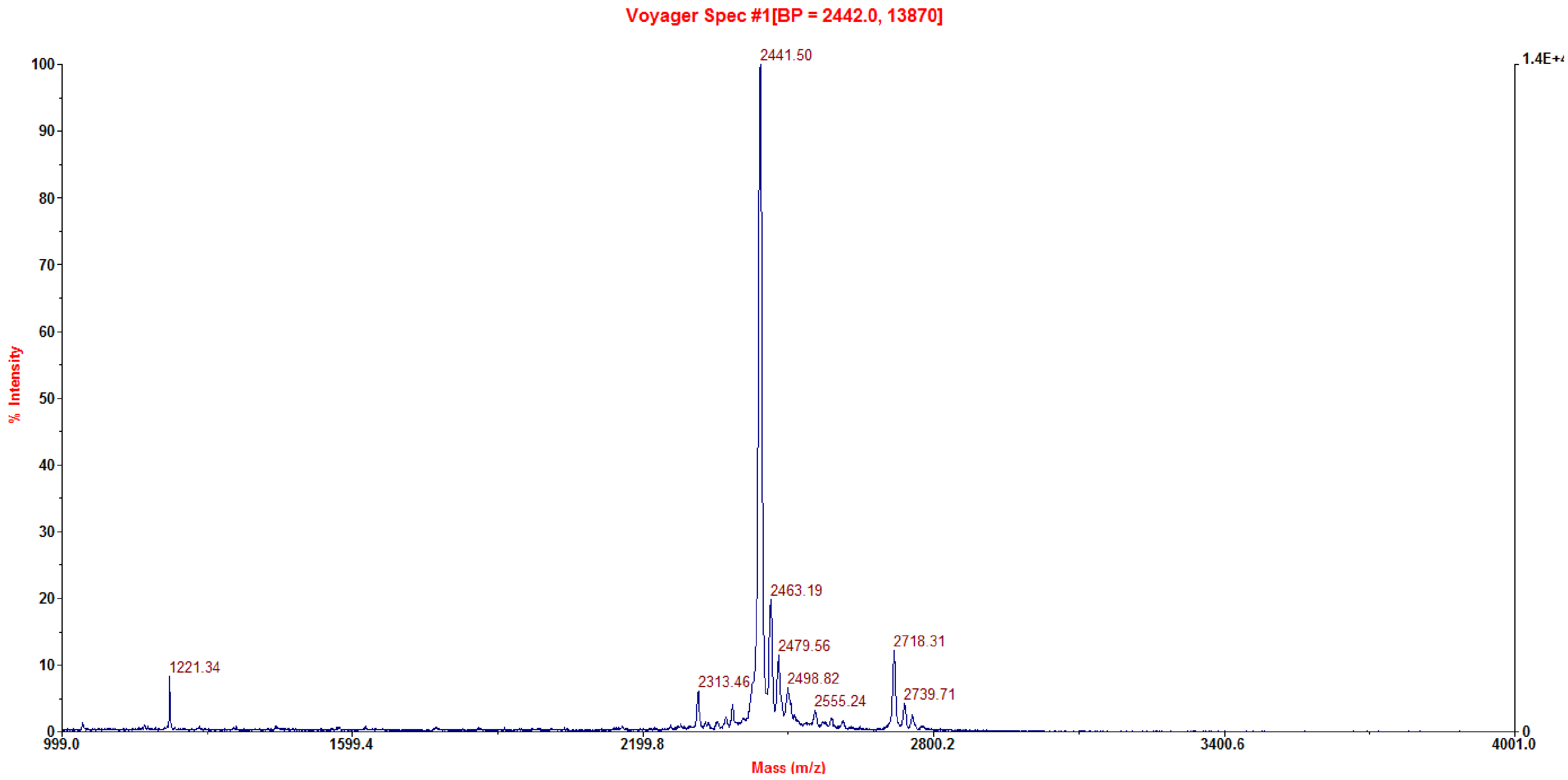

(c)

Figure S1 MALDI-TOF (Perceptive Biosystem, Bedford, MA, USA) mass spectrum of synthetic peptide (a) bombinin HL and (b) bombinin HD and (c) BHL-bombinin. In (b), the initial neutral molecule bombinin HD [M] and metal ion adducts ([M+Na]+: 1654.35Da and [M+K]+: 1669.70Da) were observed.

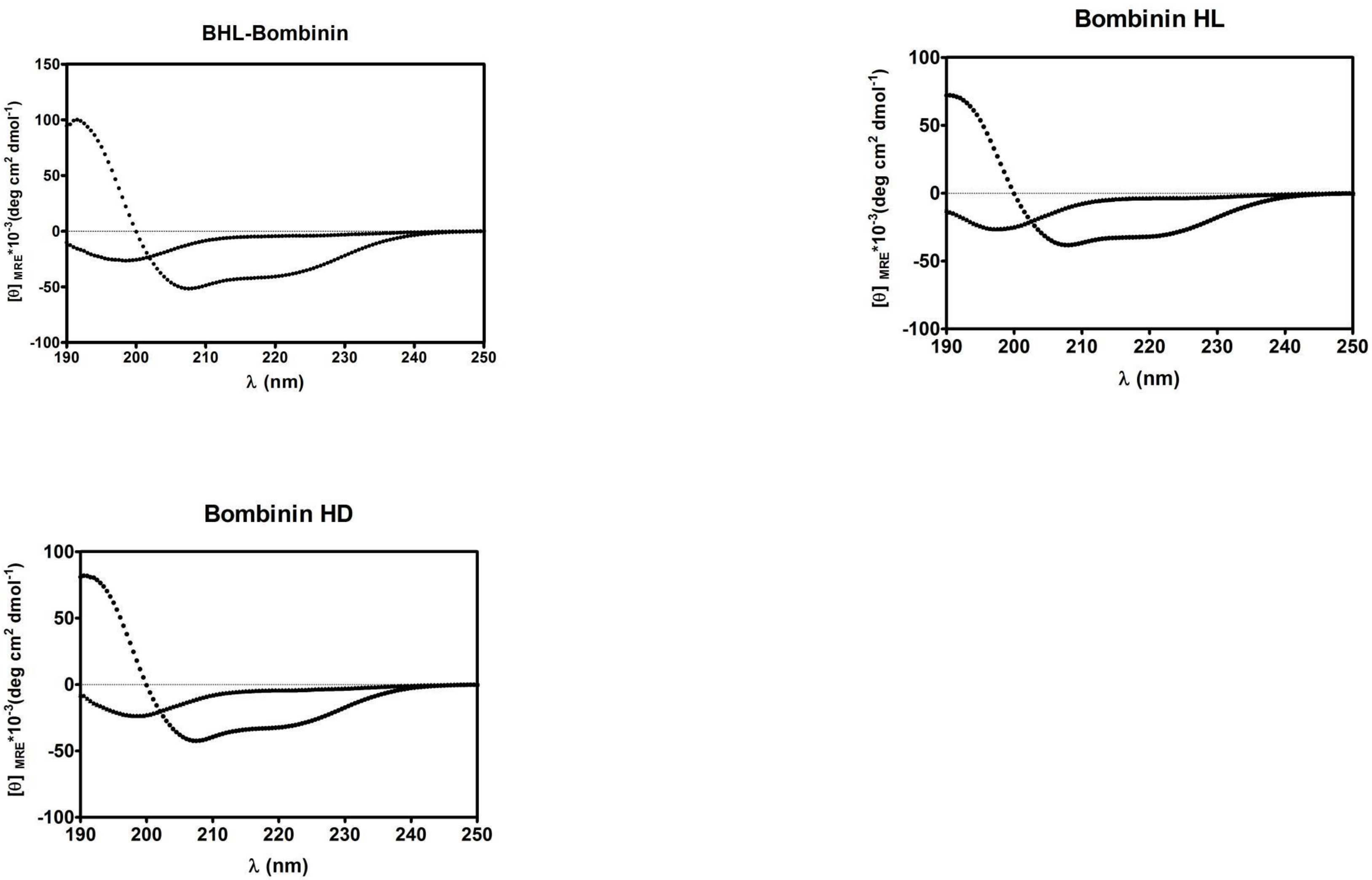

Figure S2 CD spectra of the peptides in 10 mM ammonium acetate buffer (triangles) and 50% TFE (circles). The mean residue ellipticity was plotted against wavelength. The values from three scans were calculated as average per sample. The peptide concentrations were fixed at 100 μM.

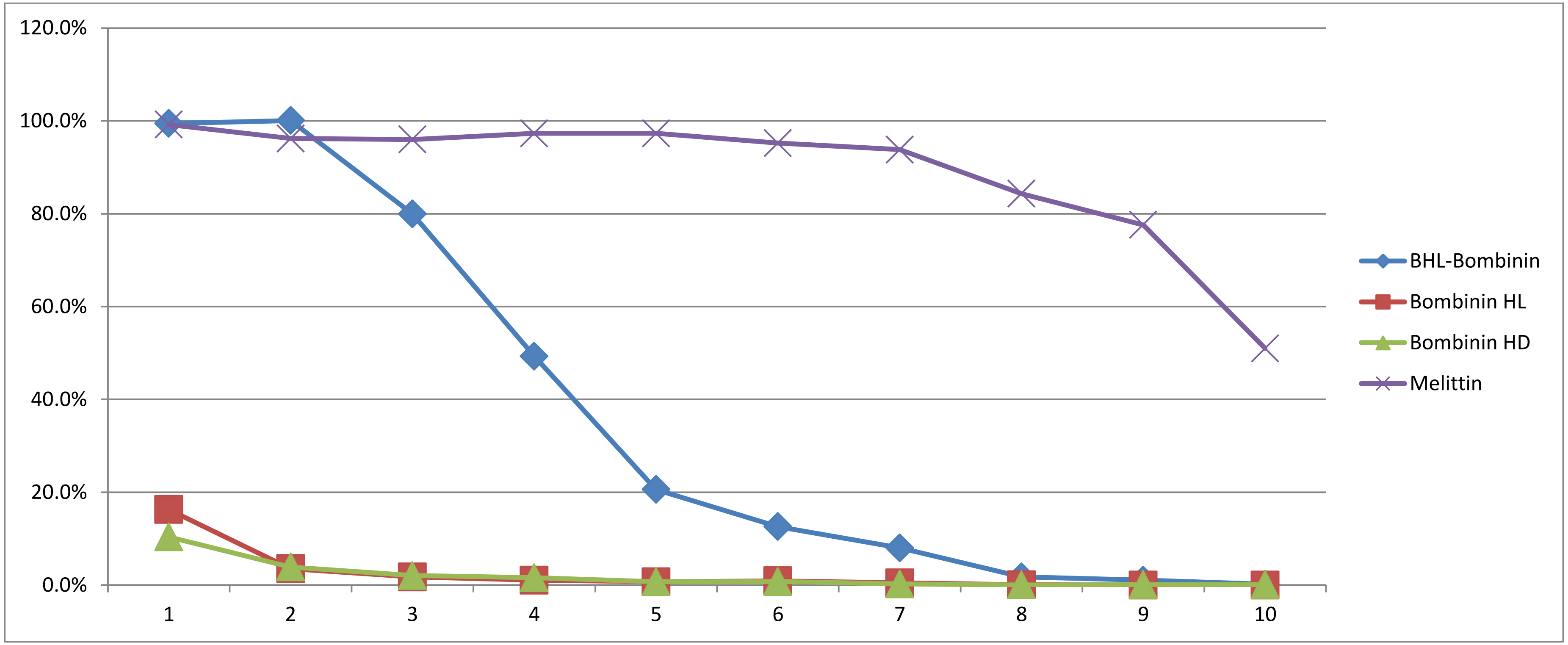

Figure S3 Haemolytic activities of BHL-bombinin (diamond), bombinin HL (square), bombinin HD (triangle) and melittin (cross) following incubation with horse erythrocytes for 2 h.

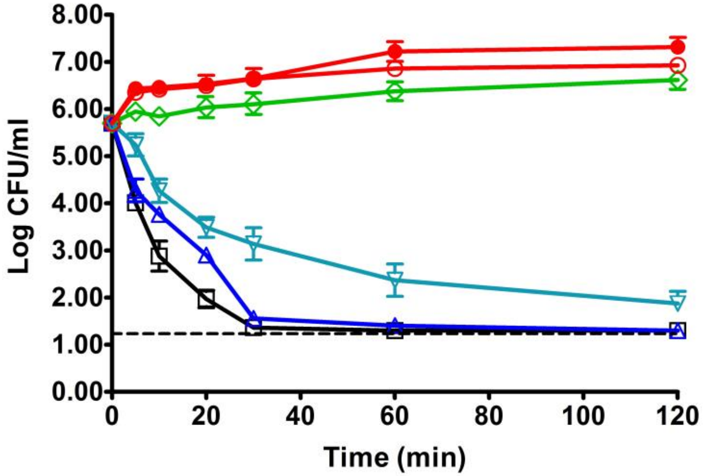
8.00
7.00
6.00
5.00
4.00
3.00
2.00
1.00
0.00
Log CFU/ml
0
20
40
60
80
100
120
Time (min)

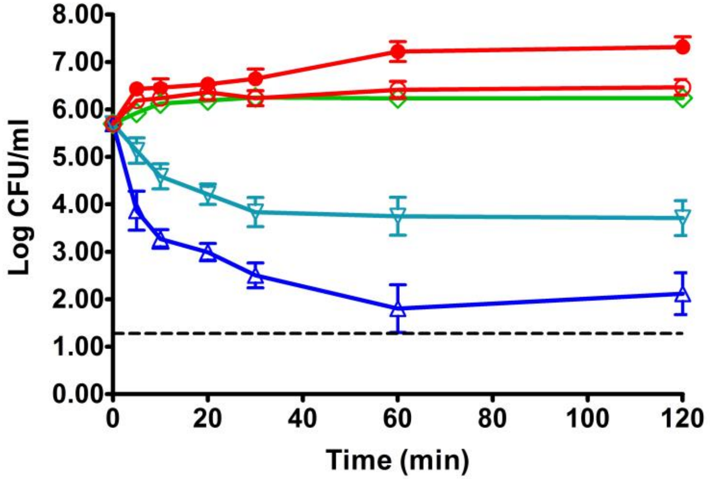
8.00
7.00
6.00
5.00
4.00
3.00
2.00
1.00
0.00
Log CFU/ml
0
20
40
60
80
100
120
Time (min)

(a) (b)

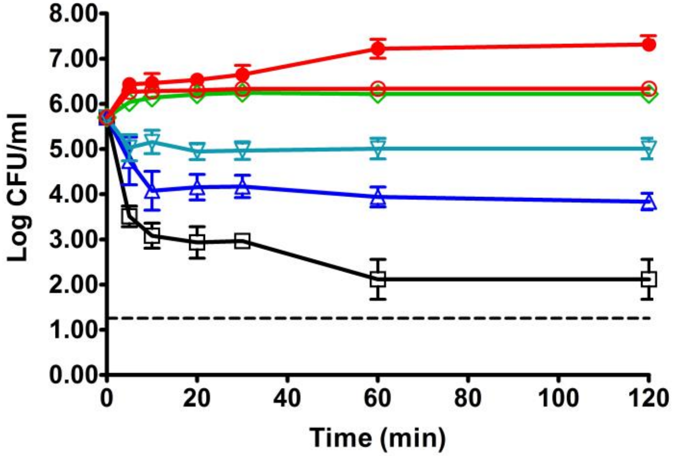

8.00
7.00
6.00
5.00
4.00
3.00
2.00
1.00
0.00
Log CFU/ml
0
20
40
60
80
100
120
Time (min)

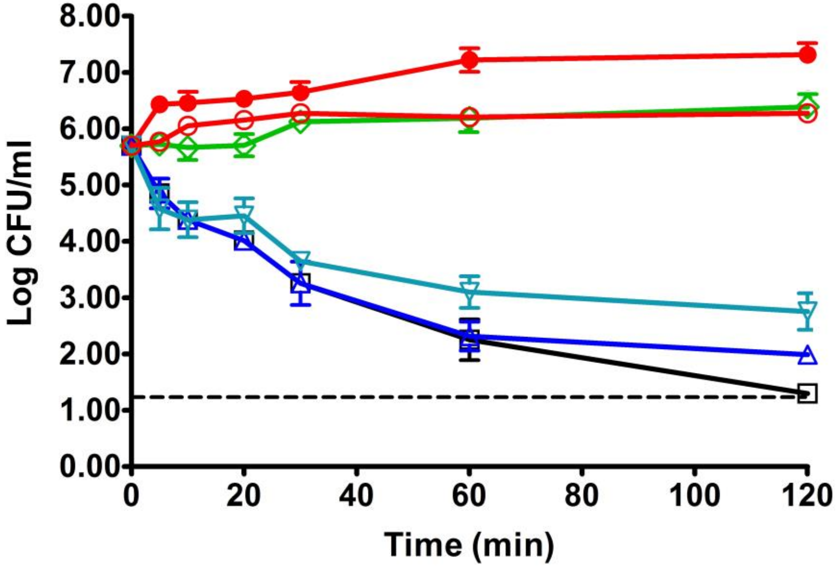

8.00
7.00
6.00
5.00
4.00
3.00
2.00
1.00
0.00
Log CFU/ml
0
20
40
60
80
100
120
Time (min)

(c)

(d)

A

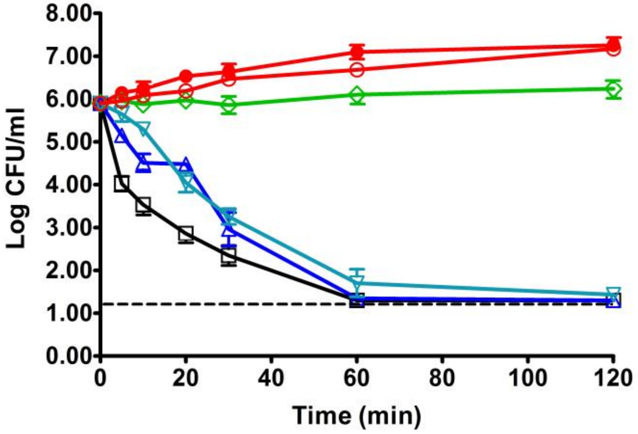
Log CFU/ml
8.00
7.00
6.00
5.00
4.00
3.00
2.00
1.00
0.00
0
20
40
60
80
100
120
Time (min)

(e)

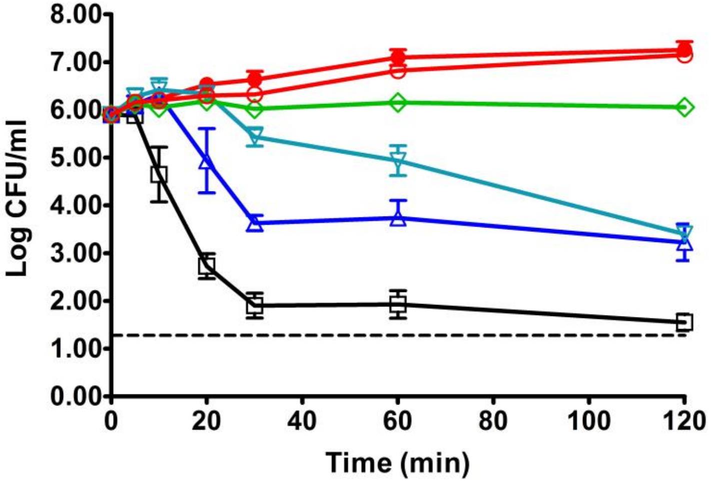
Log CFU/ml
8.00
7.00
6.00
5.00
4.00
3.00
2.00
1.00
0.00
0
20
40
60
80
100
120
Time (min)

(f)

B

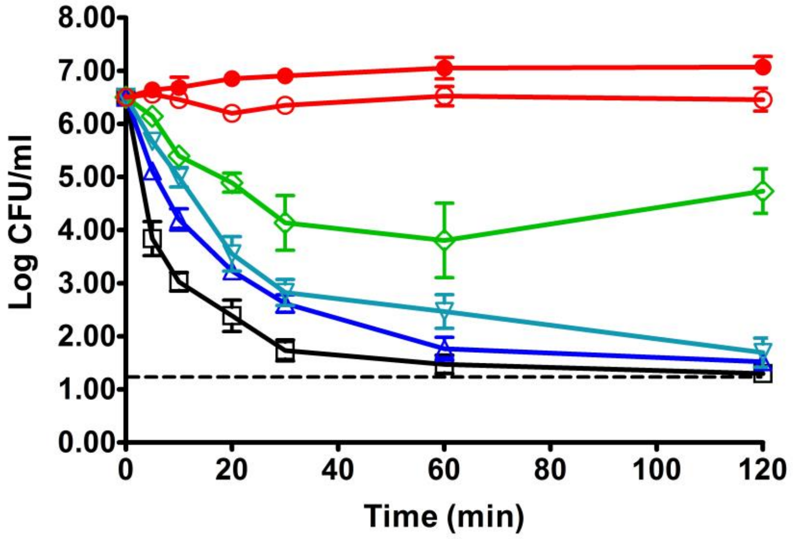

(g)

C

Figure S4 Time-killing curves of peptides and ampicillin at a series of concentrations: control (red filled circle), 0.25×MIC (red unfilled circle), 0.5×MIC (green diamond), 1×MIC (blue inverted triangle), 2×MIC (purple triangle) and 4×MIC (black square) against A: *S.aureus* [(a) BHL-bombinin (b) bombinin HL (c) bombinin HD (d) ampicillin]; B: *E.coli* [(e) BHL-bombinin (f) ampicillin]; C: *C.albicans* [(g) BHL-bombinin]. The detection limit was indicated as dashed line and the graphs were derived value of three independent trials.
